# Seroprevalence of a “new” bacterium, Simkania negevensis, in renal transplant recipients and in hemodialysis patients

**DOI:** 10.1186/s12882-017-0548-z

**Published:** 2017-04-13

**Authors:** Andrea Angeletti, Roberta Biondi, Giuseppe Battaglino, Eleonora Cremonini, Giorgia Comai, Irene Capelli, Gabriele Donati, Roberto Cevenini, Manuela Donati, Gaetano La Manna

**Affiliations:** 1 Department of Experimental, Diagnostic, Specialty Medicine, Nephrology, Dialysis, and Renal Transplant Unit, S. Orsola University Hospital, Via G. Massarenti 9, 40138 Bologna, Italy; 2 Microbiology, DIMES, University of Bologna, Bologna, Italy

**Keywords:** Kidney transplantation, Chronic hemodialysis, Simkania *negevensis*, Chronic inflammation, Infection, Hemodialysis fluid

## Abstract

**Background:**

*Simkania negevensis* is an obligate intracellular bacterium belonging to the family Simkaniaceae in the Chlamydiales order. It is considered an ubiquitous microorganism and aquatic environments may be involved as a source of infection for humans. It was just isolated in samples from domestic water supplies and from mains water supplies, like spa water or swimming pool water, confirming its ability to resist to the common chlorination treatments. Evidence indicates a possible role of the microorganism in respiratory tract infections, in gastroenteric disorders and in the pathogenesis of cardiovascular disease, furthermore it has hypothesized that it could play a role in lung transplant rejection. Prevalence and possible effects in nephrology are unknown.

**Methods:**

We examined the occurrence of *Simkania negevensis* in two differents populations, both characterized by a high susceptibility to infectious complications: 105 hemodialysis patients, 105 renal transplant recipients and 105 healthy subjects through the IgG and IgA response to *Simkania negevensis* in their sera. Serum antibodies to *Simkania negevensis* were detected by a homemade ELISA performed according to the Kahane’s protocol. Furthermore water samples from hemodialytic circuit were collected, to evaluate Simkania *negevensis* resistance to usual treatment of disinfection.

**Results:**

Our results were unexpected, showing a higher seroprevalence of antibodies against *Simkania negevensis* in the hemodialysis patients, compared to renal transplant patients (IgG 22% vs 9% - IgA 9% vs 3%). S. *negevensis* was isolated in all water samples analyzed.

**Conclusions:**

Our study detected for the first time the occurrence of *S. negevensis* in hemodialysis and in renal transplant patients. Our findings suggest that water used in hemodialysis could be one of the possible sources of S. negevensis infection, without clinical involvement risk for patients.

## Background

Chronic kidney disease (CKD) has been recognized worldwide public health problem, and the associated morbidity and mortality in patients reaching end-stage renal disease (ESRD) is constantly increasing [1, 2]. Despite the significant improvements and advanced dialysis technology [3–5], renal transplantation still remains the treatment of choice for end stage renal disease patients, and over the past decades the overall survival rate of kidney grafts has improved consistently. Moreover, because of the wide gap between organ supply and demand, there is a growing number of patients with kidney failure who inexorably accumulate on renal transplant waiting lists [6], as well as the number of renal transplant (RT) recipients with functioning grafts going to be exposed to pathogens [7]. Because of immunosuppressive therapy, infections frequently occur after kidney transplantation with greatly impact on patient morbidity and mortality. This could explain that infections are the second cause of death in renal transplant recipients, following cardiovascular diseases [8]. It is associated with an increased risk of acute cellular rejection and loss of graft function [9]. The most common infections are due to bacteria and viruses, with increasing number of parasitic infections in the last years. Infection is a common complication of hemodialytic treatment too. It has been identified as the second cause of death also among hemodialysis (HD) patients and hospitalization for infection in the HD population has increased in the last decades (2). Many of these infections are due to sepsis primarily arising from the vascular access site. *Simkania negevensis* (*S. negevensis)* is an obligate intracellular bacterium belonging to the family *Simkaniaceae* in the *Chlamydiales* order, able to survive and grow as an amoeba resisting microorganism in trophozoites and cysts of *Acanthamoeba* and other free living protozoa, which probably represent its natural reservoirs. S*. negevensis* was first defined in 1993 [10]. It was found that not only the organism was able to replicate in *Acanthamoeba*, but it was also able to survive over long periods in amoebal cysts. Since free-living amoebae such as *Acanthamoebae* may be found in many water sources, it may be that the natural source of transmission of *S. negevensis* to man is via such amoebae. A widespread human exposure to *S. negevensis* has been reported in epidemiologic studies both in healthy subjects and in association with bronchiolitis in infants, both as community acquired pneumonia and as acute exacerbation of chronic obstructive pulmonary disease in adults [11–13]. However diagnostic tools developed during the last decade will contribute to better understand more precise epidemiology and pathogenic role in pneumonia and other clinical contexts, but also to a better understanding about its distribution among healthy subjects. The seropositivity to *S. negevensis* in healthy population groups suggested the organism is a simple colonizer. However, in vitro studies have shown that *S. negevensis* can infect human cell cultures of various tissue origins, such as respiratory epithelial cells, gastrointestinal tract, genital tract and endothelial cells [14]. Because of a possible role as new pathogenic agent of lower respiratory tract infection, it was investigated among specifically immunocompromised hosts, and it was demostrated that S*. negevensis* is most prevalent in lung transplant recipients [15]. The prevalence and pathogenic potential role of S. *negevensis* in RT recipients and in HD patients is still unknown. In this prospective study we proposed to analyze the *S. negevensis* occurrence in HD and RT patients, through IgG and IgA response to *S. negevensis* in blood simple: IgA are antibodies produced by the immune system in the first phase of the infection or during any episodes of reactivation while IgG are “memory” antibodies, which appear later and remain circulating in the blood for all life from the first contact with S. *negevensis*. This investigation was prompted by previous S. *negevensis* detection in water sources and by its relatively resistance to chlorination procedures used for routine treatment of drinking water supplies [16] and Spa swimming water [17]: water samples were collected from HD course to evaluate S. *negevensis* resistance to the purifying treatment commonly performed for HD fluid.

## Methods

### Population

A total of 315 subjects were enrolled during 2013. The informed written consent was obtained from all subjects. At samples collection, RT and HD patients have been interviewed regarding respiratory symptoms and/or gastroenteric disorders; furthermore they were monitored about cardiovascular events for a follow up time of 2 years.

#### Renal transplant patients

105 RT patients, treated at Nephrology, Dialysis and Kidney Transplantation Unit S. Orsola - Malpighi University Hospital (Bologna, Italy) after performing kidney transplantation, were enrolled. The mean age was 52 years (ranging from 28 to 77 years); the mean period after transplantation was 7 years (ranging from 1 to 30 years) and the mean glomerular filtration rate was 54,6 ml/min (ranging from 22 to 76 ml/min). Blood samples were taken for physician tests.

#### Hemodialysis patients

105 chronic undergoing hemodialysis patients with no residual renal function and with no diuresis were enrolled. They came from two dialytic metropolitan centers, under the care of our medical staff. The mean age was 68 years (ranging from 28 to 77 years). Blood samples were taken for physician tests. The RT and HD patients were divided into three groups including patients from 28 to 44 years, 45 to 60 years and 61 to 77 years.

#### Control group

The control group consisted of 105 healthy people whose mean age was 58 years (ranging from 26 to 70 years). These healthy subjects were divided into three groups including subjects from 26 to 40 years, 41 to 55 years and 56 to 70 years.

### Simkania detection

Patients Sera were collected from the 105 RT and from the 105 HD and tested for the presence of IgA and/or IgG to S. *negevensis*. As controls, sera from 105 healthy subjects were used. Six months after the collection of the first serum sample, a second serum sample was collected from ten patients belonging to each of the two groups, including all patients presenting IgA and 8 patients presenting high IgG titres against S. *negevensis*. However, only one sample was collected from controls, as no IgA against S. *negevensis* was detected in these healthy subjects. S. *negevensis* Z reference strain (American Type Culture Collection VR-1471) was cultured in LLC-MK2 cells [18] and Simkania infectious elementary bodies (EBs) were purified by use of sucrose gradients [19]. Serum antibodies to S. *negevensis* EBs were detected by a homemade ELISA performed according to the Kahane’s protocol [20]. Microtiter plates were coated overnight at 4 °C with 100 Ol of EBs at the concentration of 1 Og/ml in 0.05 M carbonatebicarbonate buffer pH 9.6 and then blocked and rinsed four times with Tween 20 PBS (PBST) pH 7.4. Serum samples, diluted 1:100 in PBS-T supplemented with 5% bovine serum albumin in volumes of 100 Ol per well, were assayed in duplicate and incubated for 1 h at room temperature. Each plate assayed included replicate dilutions of two reference sera with known activity. Contents of the plates were then removed and replaced with 100 Ol of 6 M urea for 15 min. After four washes with PBS-T the plates were incubated with a 1:500 dilution of horseradish peroxidase (HRP) conjugated antibody anti-human IgG or IgA (Dako, Denmark), depending on the antibody immunoglobulin class to be detected. After incubation for 60 min at room temperature, the plates were washed and the tetramethylbenzidine substrate (TMB) (Thermo Scientific, Meridian, USA) was added. After incubation at room temperature for 10 min, the reaction was stopped by adding 3 M NaOH and the plates were read at a wavelength of 450 nm, against a reference wavelength of 630 nm. A positive sample was defined as a sample that yielded an OD450 value of at least two standard deviations above the mean of 20 negative samples. Positive sera were tested by serial dilutions to detect their antibody titre.

#### Water

A total of 4 water samples were collected from HD tap water in the same center in two different occasions: two samples from tap sited before specific water treatment of disinfection and two samples from tap sited after the specific treatment of disinfection. The presence of S*. negevensis* in the water samples was detected as previously described [17].

### Statistical analysis

Kruskal-Wallis test was used in the statistical analysis of the data: *P* values < 0.05 were considered statistically significant.

## Results

IgG and IgA response to *S. negevensis* detected by ELISA are shown in Table 1. IgG positive sera showed titres ranging from 100 to 800 and IgA positive sera from 100 to 400. All patient sera positive for IgA were also positive for IgG against *S. negevensis.*

**Table 1:** IgG and IgA antibodies to *S. negevensis* in adult patients by ELISA technique in sera

	IgG	IgA
Kidney transplant recipients	10/105 (9)	3/105 (3)
Hemodyalisis patients	23/105 (22)	9/105 (9)
Healthy subjects	32/105 (30)	0/105 (0)

Data are presented as N° of ELISA positive sera/N° of sera tested (%)

### Control group

The prevalence of IgG antibodies to S. negevensis was 30% (32/105) in sera of the healthy subjects and no specific IgA were detected in these sera.

### RT patients

The prevalence of IgG antibodies to S. *negevensis* was 9% (10/105) in sera of kidney transplant recipients. Specific IgA were detected in 3% (3/105) of the sera of RT patients.

The second serum sample, collected from the RT *S. negevensis* positive patients, six months after the collection of the first sample, did not show any significant difference related to the presence and titre of antibodies to *S. negevensis*. The average value of the number of leukocytes was 6.040/mmc (ranging from 2.000 to 13.000) in patients with no antibody to *S. negevensis* versus 7.290/mmc (ranging from 6.000 to 11.000) in patients serologically positive against *S. negevensis* (*p* < 0.043). No statistically significant results were found for other tests, even if the index of nonspecific inflammation VES was consistently with higher values in the positive than in the negative patients (Table 2).

**Table 2:** Blood tests in Renal Transplant Patients with IgA or/and IgG antibodies to S. *negevensis* positive compared to those IgA or/and IgG antibodies negative

RT patients	S. *negevensis*								Kruskal-Wallis, *p*-value
	Negative				Positive				
	Mean	Median	Min	Max	Mean	Median	Min	Max	
Hemoglobin	12.49	12.40	8	16	12.60	12.40	11	15	n.s.
GOT	16.87	16.00	6	53	14.25	14.50	9	18	n.s.
GPT	14.91	12.50	4	53	11.25	10.00	6	26	n.s.
ESR	19.95	15.00	2	63	30.40	34.00	8	51	n.s.
CRP	1.54	0.77	0.2	9.6	1.4	0.9	0.2	8.9	n.s.
Leukocytes	6.43	6.04	2	13	7.54	7.29	6	11	0.043
Neutrophils	3.79	3.54	1	9	4.75	4.73	3	7	n.s.
Lymphocytes	1.80	1.68	1	4	1.87	1.47	1	4	n.s.
Monocytes	0.48	0.47	0.2	1	0.48	0.49	0.2	1	n.s.
Eosinophils	0.15	0.1	0.1	9.5	0.17	0.18	0.1	8.1	n.s.

*GOT* glutamic oxaloacetic transaminase (mg/dl), *GPT* glutamic pyruvic transaminase (mg/dl), *ESR* erythrocyte sedimentation rate, *CRP* C-reactive proteine (mg/l). Hemoglobin values are presented as g/dl. Leukocytes, Neutrophils, Lymphocytes, Monocytes and Eosinophil values are presented as mg/dl

### HD patients

The prevalence of IgG antibodies to S. negevensis was 22% (23/105) in patients undergoing chronic hemodialysis. Specific IgA were detected in 9% (9/105) of the sera of HD patients. The second serum sample, collected from HD *S. negevensis* positive patients six months after the collection of the first sample, did not show any significant difference related to the presence and titre of antibodies to *S. negevensis*. In this population there is no difference statistically significant detectable for all physician tests between seronegative and seropositive patients against S. *negevensis*, even if the specific and non-specific inflammation tests as VES, PCR, leukocytes and neutrophils were consistently with higher values in the seropositive than in the seronegative patients (Table 3).

**Table 3:** Blood tests in Hemodialysis Patients with IgA or/and IgG antibodies to S. *negevensis* positive compared to those IgA or/and IgG antibodies negative

HD patients	S. negevensis								Kruskal Wallis, *p*-value
	Negative				Positive				
	Mean	Median	Min	Max	Mean	Median	Min	Max	
Hemoglobin	9.68	10.15	8	14.06	10.18	9.85	8	13	n.s.
GOT	17.37	12.00	4	140	13	11	6	29	n.s.
GPT	14.05	12.00	3	64	12.45	9	2	33	n.s.
ESR	40.28	37.50	2	84	48.9	47	25	101	n.s.
CRP	1.34	0.67	0.1	3.6	1.6	0.78	0.1	6	n.s.
Leukocites	6.31	6.18	2	14	8.28	6.94	3	23	n.s.
Neutrophils	3.57	3.30	1	9	5.25	4.26	2	15	n.s.
Lymphocytes	1.56	1.17	1	4	1.51	1.50	1	3	n.s.
Monocytes	0.44	0.45	0.2	1	0.43	0.43	0.2	1	n.s.
Eosinophils	0.25	0.16	0.1	1	0.28	0.19	0.1	1	n.s.

*GOT* glutamic oxaloacetic transaminase (mg/dl), *GPT* glutamic pyruvic transaminase (mg/dl), *ESR* erythrocyte sedimentation rate, *CRP* C-reactive proteine (mg/l). Hemoglobin values are presented as g/dl. Leukocytes, Neutrophils, Lymphocytes, Monocytes and Eosinophil values are presented as mg/dl

### HD and RT patients

When HD and RT patients are considered together, ESR median value in *S. negevensis* seronegative patients was 25 (ranging from 2 to 84) in comparison to the mean value of 39 (ranging from 8 to 101) in *S. negevensis* seropositive patients (*p* < 0.037). The mean value of leukocytes number was 6.090/mmc (ranging from 2.000 to 14.000) in patients serologically negative against *S. negevensis* versus 7.290/mmc (ranging from 3000 to 23000) in seropositive patients (*p* < 0.038). The average number of neutrophils was 3420 (ranging from 1.000 to 9.000) in patients that showed no antibody to *S. negevensis* in comparison to 4.500/mmc (ranging from 2.000 to 15.000) in patients that presented IgA and/or IgG antibodies to *S. negevensis* (*p* < 0.018) (Table 4).

**Table 4:** Blood tests in all patients (RT patients + HD patients) with IgA or/and IgG antibodies to S. *negevensis* positive compared to those IgA or/and IgG antibodies negative

HD and RT patients	S. *negevensis*								Kruskal-Wallis, *p*-value
	Negative				Positive				
	Mean	Median	Min	Max	Mean	Median	Min	Max	
Hemoglobin	11.21	11.80	8	36.060	11.15	11.15	8	15	n.s.
GOT	17.04	15.00	4	140	13.53	13.00	6	29	n.s.
GPT	14.61	12.00	3	64	11.95	10.00	2	33	n.s.
ESR	29.84	25.00	2	84	42.73	39.00	8	101	0.037
CRP	0.59	0.30	0.1	3.6	1.05	0.33	0.1	6	n.s.
Leukocytes	6.39	6.09	2	14	7.98	7.29	3	23	0.038
Neutrophils	3.71	3.42	1	9	5.05	4.50	2	15	0.018
Lymphocytes	1.72	1.47	1	4	1.66	1.47	1	4	n.s.
Monocytes	0.47	0.45	0.1	1	0.45	0.45	0.1	1	n.s.
Eosinophils	0.25	0.14	0.1	9.5	0.23	0.18	0.1	1	n.s.

*GOT* glutamic oxaloacetic transaminase (mg/dl), *GPT* glutamic pyruvic transaminase (mg/dl), *ESR* erythrocyte sedimentation rate, *CRP* C-reactive proteine (mg/l). Hemoglobin values are presented as g/dl. Leukocytes, Neutrophils, Lymphocytes, Monocytes and Eosinophil values are presented as mg/dl

### Hemodialysis waters

S. *negevensis* was isolated in all water samples analyzed: both from tap sited before specific water treatment and from tap sited after the specific treatment of disinfection.

### Clinical investigation

The Kruskall-Wallis test showed no correlation for diseases such as gastroenteric disorders, cardiovascular events or respiratory diseases between patients presenting IgA and/or IgG to *S. negevensis*, in comparison to seronegative HD and RT patients.

## Discussion

The present investigation evaluates for the first time the evidence of *S. negevensis* infection in two different populations: a cohort of RT patients and a cohort of HD patients. *S. negevensis* can induce different types of infection (active, persistent, cryptic) and it can affect respiratory epithelial cells, gastrointestinal tract, genital tract and endothelial cells, as demonstrated by in vitro studies [14]. *S. negevensis* virulence mechanisms may be modified inside the host cell by exchanging genetic material with other amoeba-resisting microorganism and develop virulence mechanisms that make Simkania able to survive and grow in human macrophages [21, 22].

### RT patients

*S. negevensis* IgA response was very low in the RT patients (overlapping to the complete absence of specific IgA highlighted in the general population) compared to the relative high presence of specific IgA highlighted in the HD population. At the same time, IgG seropositive appeared very low in the RT patients compared with the higher seroprevalence of specific IgG in the HD patients and in the healthy subjects. Therefore, we can speculate that immunosuppressive compulsory treatment, does not represent a risk factor for *S. negevensis* infection. Increase of leukocytes in patients serologically positive against S. *negevensis* is not a speculative results. At our knowledge, there are no reports regarding the association of S. negevensis with kidney transplant rejection, as for lung transplantation [15].

### HD patients

Higher prevalence of IgA to *S. negevensis* detected in the HD patients, in comparison with the RT patients and the healthy population, was unexpected. *S. negevensis* is a ubiquitous microorganism and aquatic environments involved as source of infection for humans, because of the large diffusion of the natural protozoan hosts in water habitats. To our knowledge, few researchers investigated the occurrence of *Simkania* in water environments, obtaining very different results in terms of frequency of detection [16, 23]. Our recent investigation detected the occurrence of *S. negevensis* in chlorinated water samples collected from swimming pool facilities, examining those classified as spa plants where a high water temperature (32–36 °C) was present [17]. In addition, samples from domestic water supplies were tested. *S. negevensis* was isolated in spa water and subsequently identified by PCR. Also the presence of *S. negevensis* in swimming pool water seems much higher compared to that of Legionella which has never been isolated from samples of inlet and pool water. This is probably due to the higher resistance of *S. negevensis* to the greater chlorine concentration in swimming pool waters, confirming that chlorine did not prevent, at the concentrations recommended for swimming pools, the survival and growth of Simkania. Moreover, *S. negevensis* was detected both in cold and hot water samples of the domestic water systems, while *Legionella* was isolated only from the hot water delivered from the showers. The HD patients represent obviously the cohort most exposed to water*.* In the present investigation, it is surprising to note that *S. negevensis* was isolated in all water samples collected, both before and after specific disinfection treatment of the HD fluid and that the forced continuous contact of the HD subjects with the treated water could be a constant source of infection. The similarity between the data of the second serum samples and the first samples collected from *S. negevensis* positive patients could be due to this fact. The Italian National Society of Nephrology has promoted the development of specific Guidelines for dialysis fluids. An optimal water treatment system should include tap water pre-treatment and a double reverse osmosis process. Every component of the system, including the delivery of the treated water to the dialysis machines, should prevent microbiological contamination of the fluid. Regular chemical and microbiological tests and a regular disinfection of the system are necessary. Treated tap water used to prepare dialysis fluid should be within the limits suggested by the European Pharmacopoeia for the water treatment system inlet and the reverse osmosis outlet. In addition dialysate, concentrate and infusion fluids must comply with the specific Pharmacopoeia limits. Frequently analysis on microbiological purity in dialysis fluid is a fundamental pre-requisite for dialysis quality. All Dialysis Unit Care should aim, as a matter, of course, to obtain an “ultra-pure” dialysate (microbial count <0.1 UFC/mL, endotoxins <0.03 U/mL). The two hemodialysis centers of the present study follow the guidelines of the Italian Society of Nephrology [24, 25]. Our findings, however, suggest that the water treatment system is not able to purify the tap water from the *S. negevensis* contamination and that treated water used in hemodialysis may be involved as a possible source of *S. negevensis* infection, thus explaining the higher prevalence of *S. negevensis* in the HD patients in comparison with RT patients. Mostly important, the forced continuous contact of the HD subjects with S. *negevensis* could represent one of the several factors, not fully known, implicated in the uremic inflammation. Inflammation and end-stage renal disease are already an old and intimately related couple. During the last decades, there has been much progress in elucidating the molecular mechanisms that lead to inflammatory reactions. Moreover, we have also learnt a lot concerning the characteristic inflammatory profile of the HD patients, which results from both retention of inflammatory mediators and increased tissue production. It is largely recognized that persistent inflammation can perturbate bone homeostasis, thus triggering vascular and arterial calcification, main predictors of cardiovascular mortality in HD population [26, 27]. Plenty of data exist on the direct involvement of the HD procedure on inflammation: the interaction of circulating monocytes with non-biocompatible membranes, the blood contact with non-sterile dialysate solution [28], the use of un-pure dialysate [29], the extent of convective transport, and the frequency and duration of dialysis [30] also may contribute to the inflammatory process. Even more recent studies have reported the possibility that infectious agents can trigger a cascade of biochemical and biological reactions, leading to inflammation. In particular, microorganisms, such as *Chlamydia pneumoniae*, have been implicated as causative or contributory factors, being associated with atherosclerosis progression [31–33]. Then S. negevensis, belonging to the family Simkaniaceae in the order Chlamydiales, could continously trigger the cascade of reactions, leading to systemic inflammation typical of HD population. Moreover, *S. negevensis*, just because similarly to Chlamydiae, is sensitive to tetracyclines and macrolides while ampicillin, penicillin G, bacitracin, cyclosporine and fluoroquinolones are not active against the microorganism [34]. The patients selected in the present study, in particular the HD patients, are generally treated with quinolone or cephalosporins as a non specific therapy in suspected infectious events (athero-venous fistula inflammation, suspect central venous catheter infection, non-specific gastric disorders, reported urinary tract disorders) since a susceptibility test indicating a specific therapy is rarely available in this infectious events. Therefore, our patients do not generally take a correct therapy for *S. negevensis*. About clinical involvement of S. *negevensis* infection, previous evidence indicates a possible role in respiratory tract infections and S*. negevensis* DNA was found in cardiovascular system, in particular *S. negevensis* genome sequences were amplified from sections of carotid artery tissue [35]. Moreover, some recent data suggest that the microorganism could be involved in non-specific gastroenteric symptoms [18]. In patients of the present study, detection of specific antibodies to *S. negevensis* did not show any association with respiratory diseases, gastroenteric disorders and/or cardiovascular events.

## Conclusion

Our study detected for the first time the occurrence of *S. negevensis* in HD and the RT patients. Unexpectedly we detected a higher IgA seroprevalence against *S.* negevensis in HD patients, than in RT. Furthermore, our findings suggest that water used in hemodialysis could be one of the possible sources of S. negevensis infection, without clinical involvement risk for patients. Further research are required to investigate the real role of water used in HD and to investigate the hypothesized role of S. *negevensis* as a trigger of systemic inflammation in HD population.

## Abbreviations

RT: Renal Transplantation
HD: Hemodialysis
S. *negevensis*: *Simkania Negevensis*
EBs: Elementary bodies
PBST: Tween 20 PBS
